# FDA approves lurbinectedin in combination with atezolizumab for extensive-stage small cell lung cancer

**DOI:** 10.1080/2162402X.2025.2584898

**Published:** 2025-11-18

**Authors:** Oliver Kepp, Guido Kroemer

**Affiliations:** a Université Paris Cité, Sorbonne Université, Inserm, Centre de Recherche des Cordeliers, Equipe labellisée par la Ligue contre le cancer, Institut Universitaire de France, Paris, France; b Université Paris-Saclay, INSERM US23/CNRS UAR 3655, Metabolomics and Cell Biology Platforms, Gustave Roussy, Villejuif, France; c Department of Biology, Institut du Cancer Paris CARPEM, Hôpital Européen Georges Pompidou, AP-HP, Paris, France

**Keywords:** IMFORTE, eIF2α, STING

## Abstract

The recent FDA approval of lurbinectedin plus atezolizumab for advanced small cell lung cancer underscores the promise of combining immunogenic cell death (ICD) inducers with PD-1/PD-L1 blockade. This synergistic strategy induces durable responses in refractory tumors and may extend immunotherapy benefits across diverse malignancies through rational ICD-checkpoint inhibitor combinations.

## Main text

The approval by the FDA of lurbinectedin in combination with atezolizumab for the treatment of advanced small cell lung cancer (SCLC) represents an important milestone in the clinical development of cancer immunotherapies. Here, a regimen designed to pair an immunogenic cell death (ICD) inducer with checkpoint inhibition (ICI) targeting the PD-1/PD-L1 axis gained regulatory endorsement. The combination of ICD with ICI holds substantial therapeutic promise, particularly for patients with immunologically “cold” malignancies that lack tumor-resident dendritic cells (DCs) and immune effectors. Moreover, it clinically validates the biological concept that ICD inducers able to prime T cell-mediated immunity can synergize with ICI to elicit durable anticancer responses.^1^

ICD is a specific form of regulated cell death in which stressed and dying tumor cells emit danger-associated molecular patterns (DAMPs) including the exposure or release of calreticulin (CALR), ATP, annexin A1, high mobility group box 1 (HMGB1) and type I interferons that engage pattern recognition receptors (PRRs) expressed on antigen-presenting cells (APCs), such as LDL-related protein 1, formyl peptide receptor-1, purinergic receptors (P2Y2 and P2RX7), Toll-like receptor 4 and type 1 IFN receptors (IFNARs), respectively. If emitted in appropriate spatial and temporal patterns, DAMPs can serve as “find-me” and “eat-me” signals for dendritic cell (DC) infiltration and antigen uptake, promote DC maturation, and thereby stimulate antigen presentation to CD8⁺ cytotoxic T lymphocytes (CTLs).^2^ Thus, ICD bridges innate and adaptive immunity, transforming cancer cell death into an *in situ* vaccine that elicits responses against tumor-associated antigens.

However, due to immunosuppressive mechanisms, including PD-1-PD-L1 signaling, in the tumor microenvironment, ICD on its own often has limited anticancer efficacy. Therefore, combining ICD inducers with T cell (re)-activating ICI provides a biologically coherent strategy to enhance antitumor immunity. Consistently, in preclinical models as well as in clinical trials, chemotherapeutic regimens based on well-established ICD inducers including anthracyclines and oxaliplatin synergize with antibodies blocking the PD-1-PD-L1 interaction to enhance T cell infiltration, delay cancer progression, and generate long-term immune memory against tumor-associated antigens.^1^ ^,^ ^3^ ^,^ ^4^

**Figure 1.**
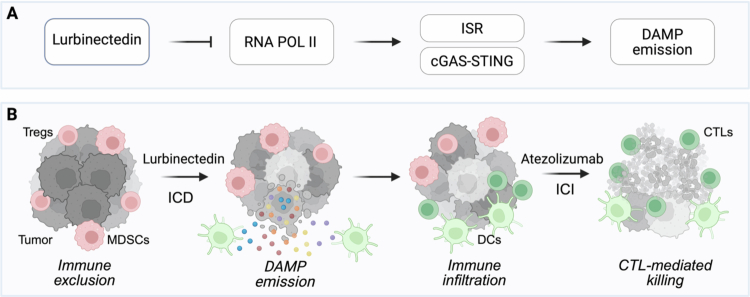
**Integrated model of immunogenic cell death (ICD) and checkpoint inhibition synergy.** (**A**) Lurbinectedin induces immunogenic cell death (ICD) in tumor cells. Mechanistically, lurbinectedin inhibits RNA polymerase II (Pol II), leading to activation of the integrated stress response (ISR) and the cGAS-STING signaling pathway. These stress responses promote the emission of danger-associated molecular patterns (DAMPs), including calreticulin (CALR), ATP, HMGB1, and type I interferons (IFNs). (**B**) Lurbinectedin-induced DAMPs engage pattern recognition receptors (PRRs) on dendritic cells (DCs), promoting their recruitment, tumor antigen uptake, and maturation. Cross-presentation of tumor antigens to CD8⁺ cytotoxic T lymphocytes (CTLs) primes adaptive anticancer immunity. Within the tumor microenvironment, PD-1/PD-L1 signaling suppresses CTL effector function; blockade of PD-L1 by the immune checkpoint inhibitor (ICI) atezolizumab restores T cell cytotoxicity and promotes durable antitumor responses. The combination of ICD induction and PD-1/PD-L1 inhibition reprograms an immunologically “cold” tumor into a “hot,” immune-responsive state, exemplified by the clinical success of lurbinectedin plus atezolizumab in small cell lung cancer (SCLC).

The combination of lurbinectedin and atezolizumab (anti-PD-L1 antibody) constitutes a prime example illustrating this strategy at the clinical level. Lurbinectedin was developed as a cytotoxic agent, which selectively inhibits transcription by covalently binding to guanine-rich promoter regions of actively transcribed genes, thereby stalling RNA polymerase II and inducing transcriptional stress.^5^ Preclinical work showed that inhibition of DNA-to-RNA transcription can generate features consistent with ICD, including the activation of the integrated stress response culminating in the phosphorylation of eukaryotic translation initiation factor 2 subunit 1 (eIF2α), which is a pathognomonic feature of ICD, the release of DAMPs and robust activation of the STING pathway leading to type I interferon secretion^6^ ^,^ ^7^) (Figure 1A). In the IMFORTE phase III trial enrolling patients with advanced SCLC whose disease had not progressed after induction therapy, maintenance therapy with lurbinectedin plus atezolizumab (anti-PD-L1 antibody) significantly prolonged progression-free survival compared with atezolizumab alone.^8^ Notably, SCLC has historically been characterized by resistance to immunotherapy, largely due to deficient antigen presentation and an intrinsically immunosuppressive tumor microenvironment.^9^ However, preclinical observations suggest that ICD-inducing therapies can overcome these hurdles and reprogram immune cold malignances into “hot” tumors heavily infiltrated by T lymphocytes, thereby rendering these cancers susceptible to subsequent ICI (10) (Figure 1B). Altogether, these proof-of-concepts advocate for broader integration of ICD inducers into immunotherapy frameworks.

In clinical practice, significant challenges remain. Many cytotoxic anticancer agents lack the ability to induce *bona fide* ICD. The capability of a drug to induce ICD depends on the induction of a specific array of premortem stress responses, the availability of a series of DAMPs, and the integrity of downstream immune sensing pathways in the host. Excessive cytotoxicity may deplete immune effector cells or damage lymphoid niches, thus compromising immune responses. Thus, it is crucial to balance immunogenic and cytotoxic effects on cancer cells while minimizing immunosuppressive side effects. Additionally, combining chemotherapeutics with immunotherapies may increase the risk of overlapping toxicities. Preclinical studies indicate that sequential strategies, i.e. administering ICD inducers before ICIs, can mitigate adverse events and improve efficacy.^10^ Integrating ICD biomarkers such as circulating DAMP levels and interferon-related gene signatures following cytotoxic therapy could further refine patient selection for ICI therapy and aid in further personalizing the treatment. Moreover, adaptive trial designs coupled with longitudinal immune monitoring will be essential to capture the complex dynamics and inform oncologists on optimal treatment options.

In sum, the approval of lurbinectedin plus atezolizumab represents a regulatory milestone that embodies a new therapeutic paradigm - one that seeks to eliminate malignant cells in an immunogenic manner, thereby generating an immune response amplified by ICI. Rather than viewing cytotoxic therapy and immunotherapy as opposing paradigms, this approach recognizes them as synergistic components of the same immunobiological continuum. Consistently employing this logic will help moving oncology closer to making tumor immune control a routine clinical reality rather than an exceptional outcome. Elucidating the full therapeutic scope of ICD induction will ultimately determine how widely this strategy can be deployed to transform the landscape of cancer immunotherapy.

## Data Availability

Data sharing is not applicable to this article as no new data were created or analyzed in this study.
